# Maturation of the HIV-1 core by a non-diffusional phase transition

**DOI:** 10.1038/ncomms6854

**Published:** 2015-01-08

**Authors:** Gabriel A. Frank, Kedar Narayan, Julian W. Bess, Gregory Q. Del Prete, Xiongwu Wu, Amy Moran, Lisa M. Hartnell, Lesley A. Earl, Jeffrey D. Lifson, Sriram Subramaniam

**Affiliations:** 1 Laboratory of Cell Biology, Center for Cancer Research, National Cancer Institute, National Institutes of Health, Bethesda, Maryland 20892, USA; 2 AIDS and Cancer Virus Program, Leidos Biomedical Research, Inc., Frederick National Laboratory, Frederick, Maryland 21702, USA; 3 Laboratory of Computational Biology, National Heart, Lung, and Blood Institute, National Institutes of Health, Bethesda, Maryland 20892, USA; 4 National Laboratory of Medicine, National Institutes of Health, Bethesda, Maryland 20892, USA

## Abstract

The formation of the HIV-1 core is the final step in the viral maturation pathway, resulting in the formation of infectious virus. Most current models for HIV-1 core formation suggest that, upon proteolytic cleavage from the immature Gag, capsid (CA) dissociates into the viral interior before reforming into the core. Here we present evidence for an alternate view of core formation by taking advantage of our serendipitous observation of large membrane-enclosed structures in HIV-1 supernatants from infected cells. Cryo-electron tomographic studies show that these structures, which contain ordered arrays of what is likely the membrane-associated matrix protein, contain multiple cores that can be captured at different stages of maturation. Our studies suggest that HIV maturation involves a non-diffusional phase transition in which the detaching layer of the cleaved CA lattice is gradually converted into a roll that ultimately forms the surface of the mature conical core.

## Abstract (web summary)

Current models of HIV maturation involve the diffusion of the cleaved capsid protein into the viral core. Here, Frank *et al*. use cryo-electron tomography to characterize HIV assembly intermediates, and propose a novel maturation mechanism involving a non-diffusional phase transition.

## Graphical abstract

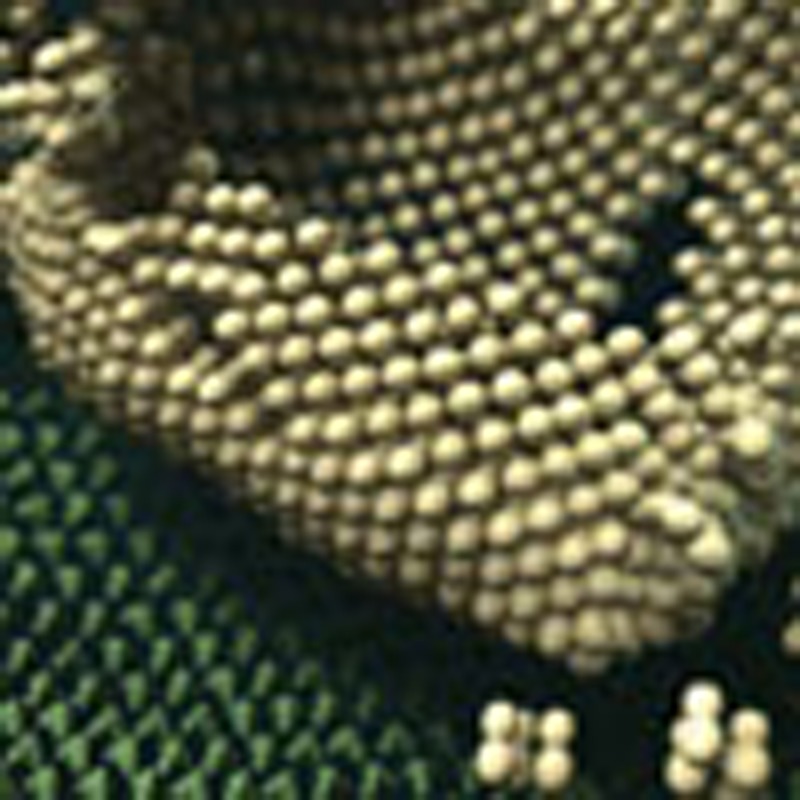

Human immunodeficiency virus 1 (HIV-1) particles transition from an immature, non-infectious form to a functionally distinct, mature infectious form. This transition requires the proteolytic cleavage of the poly-protein HIV-1 Gag, which is assembled on the inner surface of plasma membranes of infected cells. Cleavage of Gag results in a series of structural changes in the protein subunits, which give rise to the formation of a mature core that contains the viral RNA and other factors necessary for the intracellular function of the virion, and which are wrapped by a structure composed of the capsid (CA) protein. Blocking the biogenesis of mature HIV is a major strategy for drug development against HIV/AIDS^1^. Understanding the structural mechanisms involved in the conversion of the immature Gag lattice^2^,^3^ into the mature core is thus a problem of central biological interest.

Gag is a 55 kDa protein composed of several structural domains connected by short linkers^4^. The Gag domains encompass the three structural proteins of HIV: the matrix (MA), which is bound to the membrane, the CA, which eventually encloses the HIV-1 core, and the nucleocapsid (NC), which packs the HIV-1 genetic material. MA, the domain closest to the N-terminus of Gag, is responsible for targeting and binding Gag to the plasma membrane^5^,^6^ as well as for the recruitment of envelope glycoprotein (Env)^7^, which is a key for subsequent viral entry (reviewed in ref. 8). MA can form a hexagonal lattice on artificial membranes that contain the lipid Phosphatidylinositol 4,5-bisphosphate (PIP2)^9^, which is essential for MA specificity towards the plasma membrane^10^. In addition, there is indirect evidence for the presence of a similar structure *in vivo*, facilitating the recruitment of Env through non-specific interactions between the cytoplasmic tail of Env with the voids in the MA hexagonal array^11^. However, this structure has not yet been observed in electron microscopic analyses of intact HIV-1 (refs 3, 12).

During virion maturation, the internal viral structure undergoes extensive changes. These changes include the cleavage of the CA and NC domains of Gag from the MA (which remains bound to the membrane), the disappearance of the Gag lattice and the formation of the conical viral core, composed of a pseudo-hexagonal lattice of CA^13^,^14^. Most current models for assembly of the mature core suggest that the cleaved CA nucleates in a concentration-dependent manner, which perhaps in concert with binding to viral RNA results in lattice formation by polymerization, forming the conical core in a diffusion-controlled process. These nucleation and growth models also postulate that the core begins to grow at its narrow end, and stops growing once it reaches the membrane at the opposite end; thus the size of the virus itself is expected to be the primary factor that determines core size^15^. Some *in vitro* studies have suggested that the mature CA lattice structures could potentially form through a non-diffusional conversion of an intact Gag lattice^16^; however, this has been considered as an off-pathway event^17^.

Here we present findings that challenge the prevailing views and suggest an alternative model for core formation. Our observations relate to CA arrangements in both membrane-enclosed compartments found in infectious supernatants and in intact virions, and have provocative implications for HIV-1 maturation mechanisms. Instead of the nucleation-driven model, we propose that mature cores form by a direct, non-diffusional, cooperative transition from the immature Gag lattice to the mature CA lattice; that is, upon cleavage, the mature CA lattice is formed directly from the Gag lattice and gradually rolls away from the membrane, wrapping around the NC to form the core. Further, we propose an explanation for the correlation observed between the size of the HIV-1 core and the virus itself, and suggest that the factors that determine core length are controlled primarily by the size of the precursor Gag from which the mature core is generated.

## Results

### Membrane-enclosed structures with multiple cores

**Figure 1:**
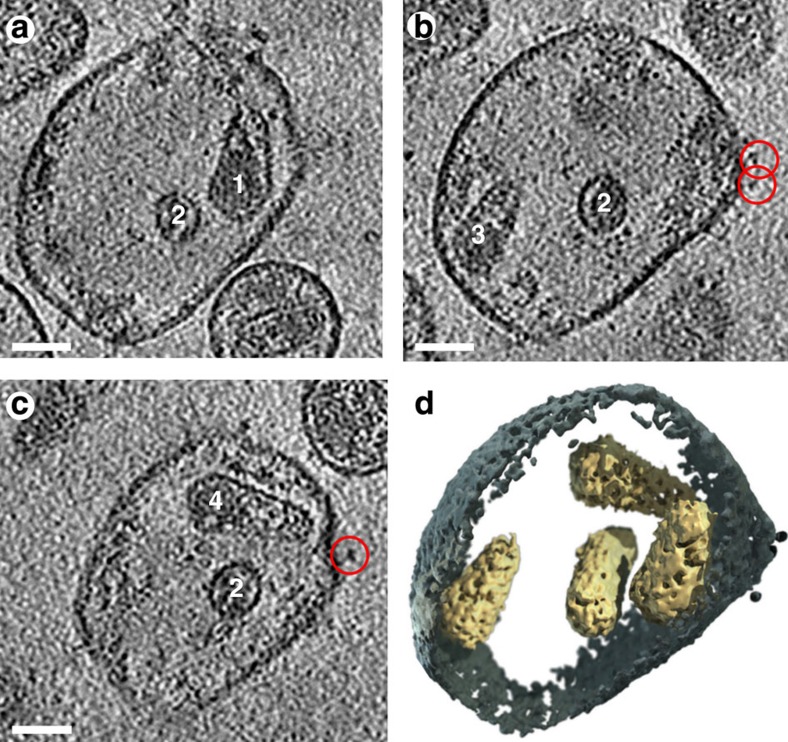
Membrane-enclosed structure containing multiple cores. (**a**–**c**) Slices through a tomogram of a typical multi-core membrane-enclosed structure found in the supernatant of a HIV-1 BaL producing T-cell line culture. Each core is designated with the same digit in all slices. Envelope glycoproteins decorating the outer membrane are circled. (**d**) 3D rendering of the tomogram in **a**–**c**. Cores 1, 3 and 4 lay close to the membrane along their sides. Core 2 floats in the centre of the membrane-enclosed volume. Scale bars, 50 nm.

We used cryo-electron tomography to analyze the morphology of viruses and other membrane-enclosed structures present in the supernatants of HIV-1-infected cells. In culture supernatants collected from SupT1-CCR5 cells chronically infected with HIV-1 BaL, we observed a small, but highly reproducible population of membrane-enclosed structures with Env displayed on the membrane surface, encasing a variable number of mature HIV-1 cores (Fig. 1). These membrane-enclosed structures are distinct from typical virions both in terms of their size and the number of cores they contain. While typical mature HIV-1 particles are ~120 to ~220 nm wide, and for the most part contain a single conical core, these membrane-enclosed structures are ~200 to ~800 nm wide, containing numerous mature cores in their interior. Numerous examples of these objects were analyzed from the tomographic data; scatter plots depicting the distribution of the number of cores per object and the estimated membrane surface area of the objects we analyzed are displayed in Supplementary Fig. 1. Some of these cores are attached at one edge to the membrane, while others are freely dispersed in the lumen of the compartment (Fig. 1). In addition, areas displaying highly curved membrane with clear immature Gag structure were also found, suggesting that the cores can form within these membrane-enclosed structures (Supplementary Fig. 1). The presence of normal-sized cores that are not in contact with the membrane further indicates that the core formation need not require the presence of membrane at both ends of the core. The implication that the dimensions of the viral envelope membrane do not dictate the size of the mature core is a shift in the conventional paradigm for the mechanism of core formation^15^,^18^.

To exclude the possibility that these objects may have somehow originated from Env-mediated fusion of multiple mature virus particles into a single structure, or from processing or fixation steps required for working with infectious virus, we carried out tomographic studies of Env^neg^ HIV-1 (a variant of HIV-1 NL4-3 that lacks the gene for Env and is thus non-infectious). Despite the absence of Env, membrane-enclosed structures containing multiple cores were present, indicating that both the wild-type and mutant strains of NL4-3 can produce these structures.

**Figure 2:**
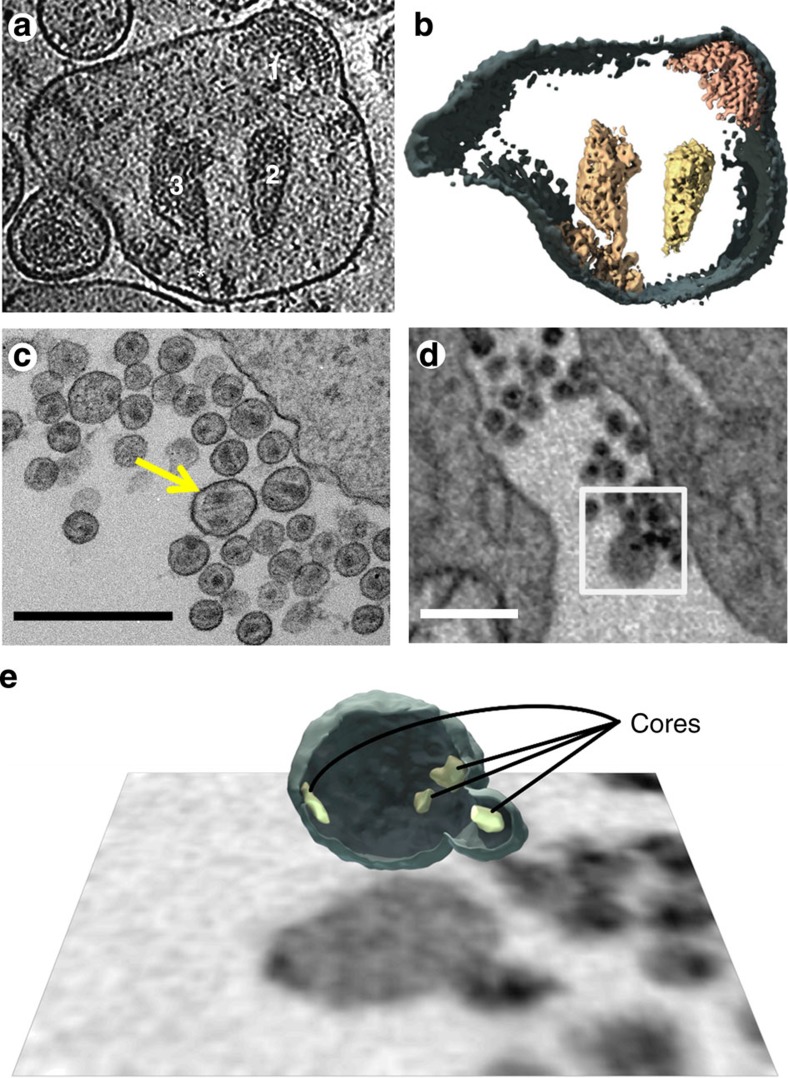
Multi-core membrane-enclosed structures do not arise through fusion or sample processing. (**a**) A tomographic slice of a multi-core structure from the Env^neg^ strain. Scale bar, 50 nm. (**b**) 3D rendering of the structure in **a** depicting an immature Gag assembly (1) and two mature cores (2, 3). Core 2 is detached from the membrane. Core 3 has an aberrant, incomplete sheet structure. (**c**–**e**) Multi-core structure found in a cell culture sample. (**c**) TEM shows regular virions and a structure with two cores, which are not in contact with the membrane at the wide end. Scale bar, 500 nm. (**d**) A slice through a multi-core structure and regular virions near a cell obtained by FIB-SEM. Scale bar, 500 nm. (**e**) 3D visualization shows three cores inside the structure from **d**.

As in the case of HIV-1 BaL, some of the Env^neg^ multi-core membrane-enclosed structures still had immature Gag assemblies attached to the inner leaflet of highly curved regions of the membrane (Fig. 2a). Three-dimensional (3D) visualization of the multi-core containing structures from Env^neg^ HIV-1 NL4-3 also shows that, as with HIV-1 BaL above, some cores lack any apparent contact with the membrane (Fig. 2b). There is also evidence for cores that are still nascent and attached to the membrane, and for cores that appear to display sheet-like shapes (Fig. 2a,b). The presence of the areas of immature Gag and incomplete cores suggests that the formation of the multi-core membrane-enclosed structures occurs prior to core maturation, and allows us to investigate the process of core formation. One puzzling question is why these multi-core containing structures appear to have eluded observation in all of our previous tomographic analyses of HIV-1 BaL virus in the context of determination of trimeric Env structure by electron tomography and sub-volume averaging. Upon re-examination, we found the remnants of these membrane-enclosed structures in each of our previous tomographic data sets; the reason these structures were previously unremarked is that the use of Aldrithiol-2 for virus inactivation, which preserves envelope glycoprotein structure and function, can lead to disulfide crosslinking of CA protein subunits, altering the ultrastructural morphology of viral cores. These structures were consequently treated as background impurities in the preparation. The cores are clearly visible in the data we present here because the methods for virus preparation do not involve Aldrithiol-2 treatment and thus loss of the mature core structure.

To evaluate the possibility that the membrane-enclosed structures containing multiple cores might be an artifact of high-speed centrifugation or other steps involved in virus purification, we imaged intact cell pellets not subjected to the physical manipulations involved in virus purification. Resin-embedded samples of infected cell culture were subjected to standard transmission electron microscopy (TEM) imaging of thin sections, as well as imaging by focused ion beam scanning electron microscopy (FIB-SEM). TEM imaging of the sample shows the presence of viral particles containing more than one core; however, due to the 2D nature of this technique, few (if any) of the particles are cut precisely through the centre, making analysis of the size of the particles and the position of the cores within the particles problematic. However, examples of large particles with more than one core were visible (Fig. 2c). Imaging of the sample by FIB-SEM, however, enables visualization of the ultrastructure of the entire cell as well as membrane compartments present inside and outside of the cell in 3D. Importantly, because FIB-SEM methods enable us to take snapshots of all of the contents present in the cell suspensions, we can assess whether the multi-core structures are present before any biochemical processing steps are initiated. Inspection of slices through the 3D volume of cells infected with HIV-1 BaL (Fig. 2d), and the associated segmented volume (Fig. 2e), demonstrates that the large multi-core structures are also present in the original infected cultures. Even though these results do not establish their biological function, they demonstrate that the multi-core entities did not arise as a result of biochemical processing procedures used for virus preparation. In addition, we measured the surface area per core of numerous multi-core containing structures and normal virions (Supplementary Fig. 1d). The majority of multi-core structures have a greater proportion of membrane area per core, as compared with single core virions. If we make the assumption that the predominant fusion event that could generate multi-core structures would have to be between virions, one would expect that the ratio of membrane surface area to cores would remain constant between single core virions and the multi-core structures. Our results show this is not the case, leading to the conclusion that formation of the multi-core structures cannot be explained by a mechanism that invokes simple virion fusion as its origin.

Taken together, our findings indicate that the formation of multi-core entities is not the result of fusion of pre-formed mature virions or an artifact of virus purification. The presence of cores with the same general size and shape as those in normal virions leads us to conclude that the dimensions of the membrane-enclosed structure do not directly dictate core length, as hypothesized in membrane-restricted nucleation and growth models^15^,^17^,^18^,^19^. The presence of normal cores inside these membrane-enclosed structures challenges current models for HIV maturation and provides a unique opportunity to explore the underlying physical mechanisms and constraints that are relevant to core formation.

### Visualization of the MA lattice

**Figure 3:**
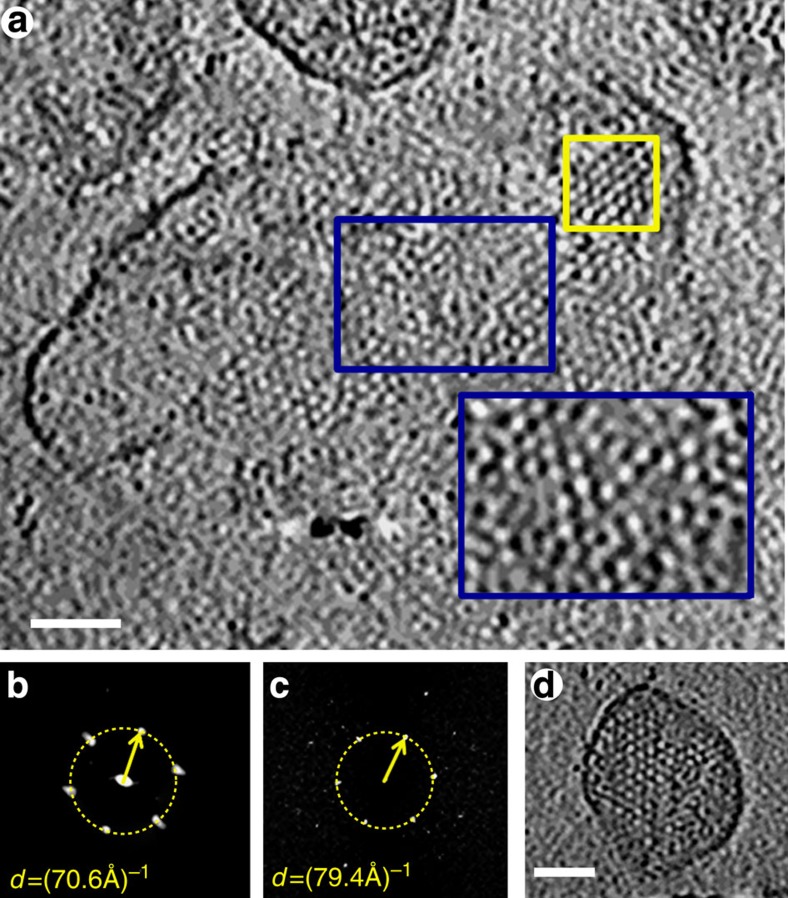
Comparison between the disordered matrix (MA) and Gag lattices. (**a**) A grazing slice through the membrane of the multi-core membrane-enclosed structure illustrated in Fig. 2a. This slice provides a top view of the Gag lattice located at the upper right corner of the panel (yellow rectangle). The inset in **a** depicts the MA lattice bound to the membrane. This slice is located 3.3 nm above the region marked by the blue rectangle (inset). (**b**,**c**) Power spectra of Gag and MA lattices are displayed in **b** and **c**, respectively. The distance of the 1st order peaks of the power spectra from the origin are marked with yellow circles and arrows, which when multiplied by (cos 30°)^−1^ provide an estimate of the corresponding lattice dimensions (~8 nm and ~9 nm, respectively). The presented power spectra were calculated from the regions marked by yellow rectangles in **a** of this figure and in Fig. 4b (**d**) Slice through a tomogram of a regular virion with flat membrane displaying MA lattice near the membrane. Scale bars in **a** and **d** are 50 nm. Inset scale bar, 25 nm.

**Figure 4:**
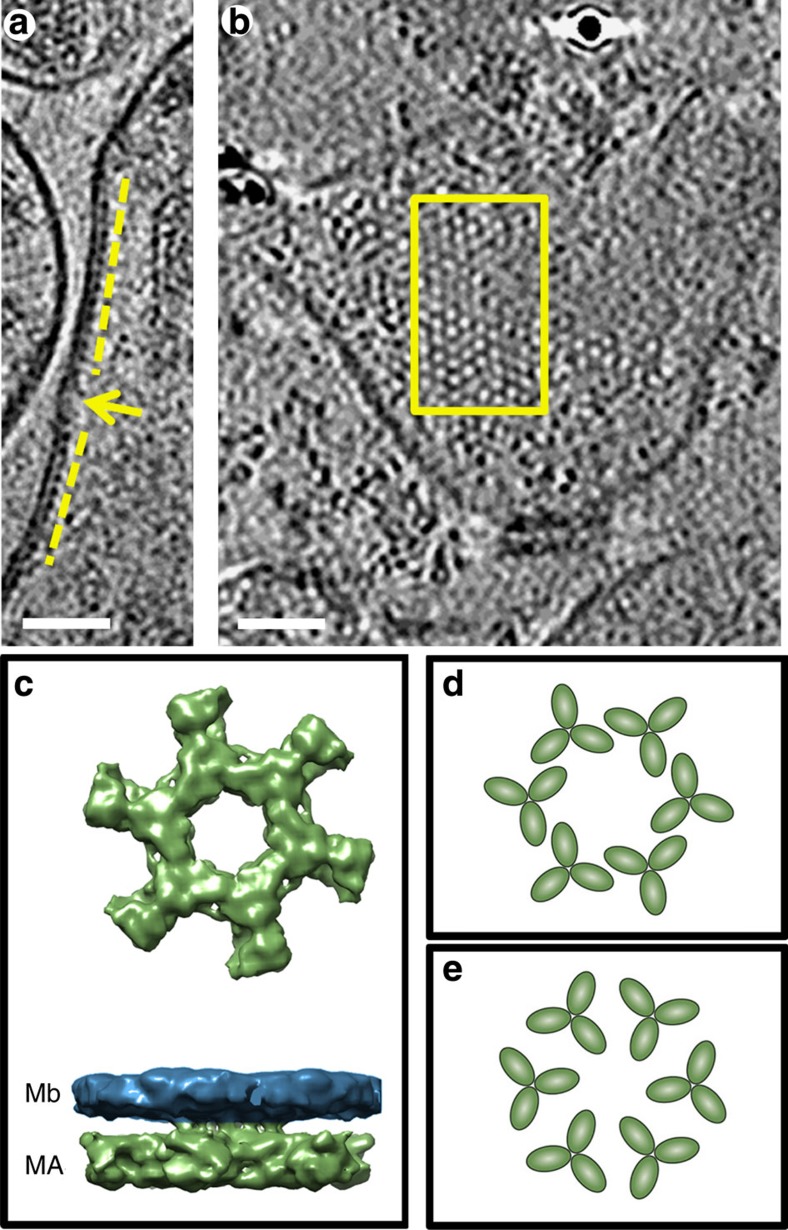
The structure of the native MA lattice. (**a**) A side view of two MA lattice structures (yellow line) near the membrane of a multi-core structure. The lattice is found near a low curvature region of the membrane and is disrupted by a region where the curvature is higher (arrow). (**b**) A grazing slice through the membrane of a multi-core structure, depicting an extended MA lattice. Scale bar, 50 nm. (**c**) Bottom (from the inner side of the membrane) and side views of the map resulting from sub-tomogram averaging of the MA lattice (with the membrane density removed from the bottom view map) showing a web-like hexagonal structure with large central voids (~6 nm). (**d**) Model for arrangement of MA trimers that could encompass the central void as measured from **c**. (**e**) Previously proposed model for MA trimer arrangement^9^ that allows for smaller voids.

**Figure 5:**
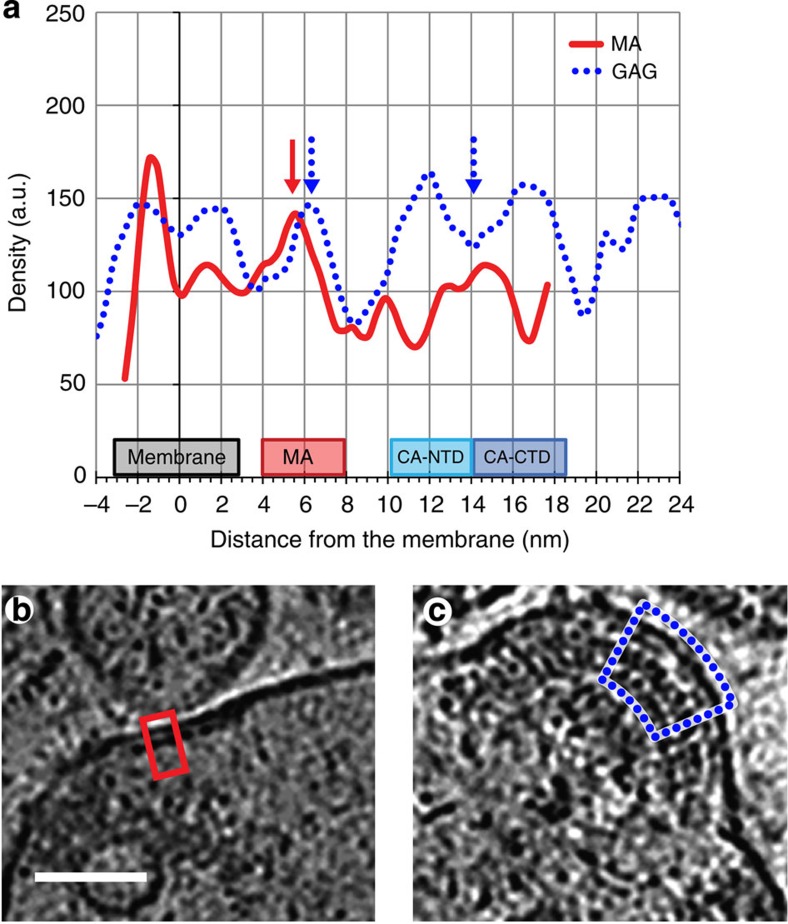
Average electron density of MA and Gag lattices perpendicular to the membrane. The MA layer of both structures (two leftmost arrows) is positioned ~4.5 nm from the inner leaflet of the membrane (5.5–6.3 nm from the centre of the membrane). The rightmost arrow points to the low-density region between N-terminal domain and the C-terminal domains of the immature CA layer in the Gag lattice. (**b**,**c**) The areas selected for the calculation of the density profiles of the MA and Gag are marked with red and dotted blue lines, respectively. Both panels are at the same scales, scale bar, 50 nm.

During virion maturation, HIV-1 protease cleaves CA subunits from the Gag assembly, leaving MA attached to the membrane. As mentioned above, the MA lattice has been directly visualized on PIP2-containing artificial membranes^9^, but has not yet been directly observed in intact virions. We observed numerous examples of the MA lattice in the multi-core structures (Figs 3a and 4a,b). A face-on tomographic slice (~6 nm below the membrane) of an Env^neg^ multi-core structure shows that the MA layer is arranged in a hexagonal lattice (Fig. 4b). These hexagonal patches were often found to be disordered (Fig. 3a), with gaps in packing. The MA lattice could be distinguished easily from the immature CA lattice of the Gag (Fig. 4b), as the two lattices are located at different heights from the membrane (Fig. 5). In addition, the two structures have different lattice parameters, as measured from the power spectra of the lattices, with the MA lattice (9±1 nm) systematically larger by ~12% than that observed for immature Gag (8±2 nm). This relative difference in packing was further confirmed by measurements of the lattice parameters when both immature CA and MA lattices were present simultaneously in the same multi-core compartment (Fig. 3). Our measurements for the immature CA lattice parameters are consistent with those observed in previous structural studies of immature HIV-1 and other lentiviruses^3^,^20^. The lattice parameters for MA packing in the compartments are also similar to the values reported for the lattice of purified MA formed on artificial membranes containing PIP2 (ref. 9).

The presence of these MA lattices strongly correlates with relatively flat areas of the membrane on the multi-core membrane-enclosed structures. In addition, examples of MA lattice were occasionally visible on normal-sized virions that had flattened membrane areas (Fig. 3d). Our identification of this density as MA lattice was confirmed by measuring the lattice parameters of these structures. These observations suggest that it is the ability of the multi-core containing structures (which have a much larger surface area and thus less pronounced membrane curvature than normal-sized virions) to form flat membrane areas that allows for the presence of the extended MA lattices shown here.

Sub-volume averaging of the MA layer allows better visualization of the packing arrangement by increasing the signal-to-noise ratio. An averaged view shows that the MA lattice lies about 6.3 nm below the membrane (Fig. 4c), which is in agreement with measurements of MA distance from the lipid bilayer obtained by averaging the electron density perpendicular to the membrane (Fig. 5)^3^,^21^. The lattice has large hexagonal voids at the centre (Fig. 4c), which are ~6 nm from edge to edge. This organization of MA in this native context is distinct from the more tightly packed arrangement (~4.3 nm diameter voids) observed under the *in vitro* conditions of the artificial lipid membrane^9^. We propose that individual MA trimers^22^,^23^ may fit into the structure with interactions near or at the tips (Fig. 4d), which would accommodate the large voids we observed, in contrast to the arrangement proposed for the MA lattice on artificial membranes (Fig. 4e).

### Intermediate stages of core formation

**Figure 6:**
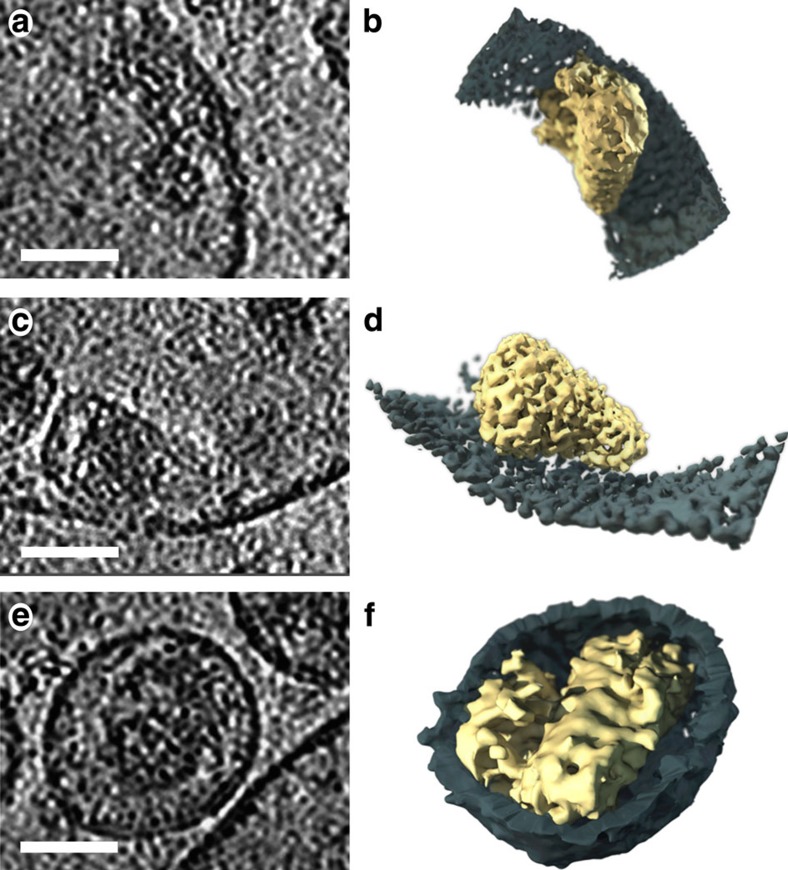
CA sheets roll to form cores. (**a**) A slice through a tomogram showing the CA layer detaching from the membrane of a multi-core membrane-enclosed structure and wrapping around the interior of the core. (**b**) A 3D rendering of the core in **a**. (**c**) A slice through a tomogram of a core at a late stage of formation. The core is fully formed but still attached to the membrane. (**d**) 3D rendering of the core in **c**. (**e**) A slice through a tomogram, showing a section perpendicular to the long axis of a forming core inside a regular size virion. A sheet of CA is connected to the virion envelope on one side and is curling around the core towards the centre of the virion. (**f**) A 3D rendering of the core in **e**. All scale bars, 50 nm.

The Gag assemblies (Fig. 2, Supplementary Fig. 1), MA lattices (Figs 3 and 4) and freely floating mature cores (Figs 1 and 2) observed above likely represent snapshots of the very early and final stages of the maturation process. In addition, several additional distinct transitional stages were also observed upon inspection of collected tomograms. In one intermediate stage, the sheet of membrane-associated CA was found to be contiguous to a region detached from the membrane and rolled partially into a mature core-like structure (Fig. 6a,b). A second intermediate, representing a late stage in the core formation process, showed nearly complete cores attached to the membrane along one side (rather than at one end, as would be expected from current core formation models; Fig. 6c,d). We also observed what is likely an early stage of the process: one can see what appears to be immature Gag lattice on the membrane present in a contiguous structure with mature CA lattice pulling away from the membrane (Supplementary Fig. 1a). For this figure we assigned the thicker protein layer and the visibly thinner layer that curls away from the membrane to Gag and CA lattices, respectively, based on established morphological markers^17^. None of these intermediate stages can be explained based on current models for core formation, which do not predict either the existence of partially rolled intermediates^15^,^17^,^18^ or the presence of cores attached to the membrane along their sides^19^. Instead, current models posit that the mature core begins to grow at its narrow end from nucleation of CA units on one face of the membrane and stops growing when it reaches the boundary of the membrane on the opposite side.

It is formally possible that the structures we observed in the multi-core containing objects were ‘abortive’ efforts at core formation unique to these objects. To determine whether the cores at intermediate stages were also present in normal virions, we re-examined the data sets and observed that membrane-bound cores that are in the process of formation into the final conical shape are also observed in regular-sized virions (one example is presented in Fig. 6e,f, and seven additional examples are documented in Supplementary Fig. 2b–h).

If the core is indeed formed by the non-diffusional phase transition of immature to mature CA lattice that we propose, one would expect to find that the stresses and deformations inherent in this process result in defects in the mature CA lattice. Such defects, which have been noted previously^19^, are visible in tomograms of most mature cores (Supplementary Fig. 3). On occasion, significant portions of the surface are absent, although the packing of CA on the remainder of the core appears to be intact (Supplementary Fig. 3e,f), with clear visualization of the canonical pentameric and hexameric geometries.

## Discussion

In this study, we have exploited our observation of membrane-enclosed structures containing multiple HIV-1 cores in specimens from HIV-1-infected cultures to provide insights into mechanisms that may be relevant to the formation of native HIV-1 virions. These membrane-enclosed structures, which are larger than virions, occur independently of fusion, and are not a product of procedures involved in sample preparation, appear to be formed prior to Gag maturation, as they include regions with clearly visible patches of immature Gag. The characteristics of these structures allow us to postulate a series of events in HIV-1 assembly and maturation that were difficult to understand in the context of normal virogenesis. Moreover, many of the structural features observed in these membrane-enclosed structures can be found upon re-examination of typical virions prepared from the supernatants of infected cultures (Fig. 6e,f, Supplementary Fig. 2), suggesting that the cores likely mature by a similar mechanism in virions and in the large membrane-enclosed structures.

**Figure 7:**
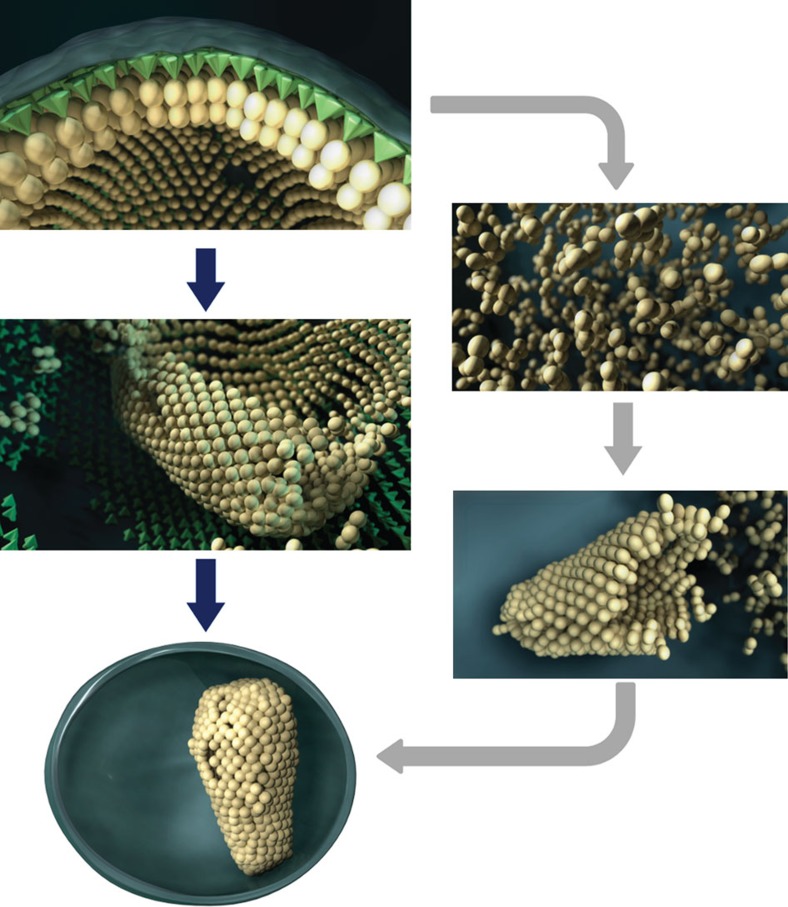
Models for core formation. (Top) In an immature virion, Gag lattice is arranged beneath the viral membrane. (Right) In the classic model, proteolytic cleavage releases the CA to the viral lumen. CA monomers then nucleate and grow in a diffusion-controlled process, closing off when the opposing membrane is reached. (Left) In our model, CA layer is released by proteolytic cleavage and rolls while transforming to the mature lattice arrangement, until the core is fully formed.

In Fig. 7, we present a schematic comparison of the model we propose here for core formation, and distinguish it from currently accepted models for HIV-1 maturation. A common feature of currently accepted models is that the release of CA monomers into the interior of the virus by proteolytic cleavage results in the nucleation of a conical core starting at one end of the membrane and stopping when it reaches the other end. In contrast, in our model, we are proposing that CA detaches as a largely intact layer and transitions into the mature lattice structure (Supplementary Movie 1), as has been suggested by *in vitro* studies^16^.

Implicit to our model is the idea that MA facilitates the assembly of Gag on nearly flat regions of the cell membrane. After MA assembles on the cellular membrane and Env is recruited to the site of the MA lattice, the CA domains coalesce into the immature Gag lattice. Our measurements indicate that the immature CA lattice is significantly tighter than the MA lattice; this mismatch may promote the curvature and budding of the nascent virion. The lattice parameter of the emerging immature CA lattice is ~8 nm, ~12% smaller than that of the MA lattice. The CA layer is also positioned ~8 nm to the interior of the MA layer. Although MA is not absolutely required for virion formation^24^, consideration of just these two geometrical constraints (in packing and axial position) results in a prediction of a packing sphere with a diameter of 140–160 nm, remarkably close to what is observed for immature and mature particles of HIV-1 and most other retroviruses^25^.

Classic nucleation and growth models for core formation predict that the diameter of the viral membrane dictates the length of the cores^15^,^18^. However, rather than the elongated tubes, one would expect from these models (similar to the tubular CA structures found as a result of *in vitro* oligomerization of CA^26^,^27^) the size of the cores formed inside the multi-core containing structures is predominantly similar to the size of the cores found in regular virions. Instead, we propose that it is solely the area of the budding virion that determines the size of the available CA lattice and consequently the final length of mature cores. The sites on the membrane-enclosed structures that resemble budding virions display a curvature and area with dimensions similar to that of native virions. As a result, based on our model, these sites are expected to produce similar sized cores. Our model for core formation also supposes that, following cleavage, the CA sheet must curl away from the membrane as an intact layer, which can explain the potent trans-dominant effect of uncleavable Gag on infectivity^28^,^29^. Lee *et al*.^28^,^29^,^30^ demonstrated that the presence of just 4% Gag with an uncleavable MA–CA linker resulted in a decline of >50% in infectivity, a finding that is not easily explained by models that invoke core formation by nucleation of released CA monomers. Furthermore, similar experiments with Gag with uncleavable CA–Spacer Peptide 1 (CA–SP1) or SP1–NC had only a modest effect on infectivity. These results are, however, completely consistent with our model, because the presence of even a few Gag subunits unable to cleave MA from CA that remain attached to the membrane would preclude CA lattice sheet detachment, thus inhibiting core formation and leading to a disproportionate drop in infectivity.

The transition of the CA from the immature to the mature conformation *in situ* requires large rearrangements of local protein–protein contacts along the interface between the immature and the already mature lattices^20^, and will likely be accompanied by large local strains in the lattice. It is also implicit in our model that the deformation and curling of the CA sheet, which ultimately allows the formation of the core, occurs as a direct result of the conformational changes of the CA that are triggered by Gag cleavage. Indeed, as the proteolytic sites flanking CA are cleaved, the forces stabilizing the immature lattice disappear. The release of the constraints enforcing the immature lattice structure allows a structural transition of the CA proteins^3^,^16^,^31^,^32^, which in turn enforces a wider spacing between the lattice subunits and thus a larger spacing of the mature lattice. We further suggest that, given that the non-diffusional phase transition takes place *in situ* and prior to the CA lattice dissociation, even if both processes (the non-diffusional transition and the CA dissociation followed by *de novo* nucleation and growth) occur *in vivo*, the non-diffusional phase transition would be heavily favoured kinetically.

Consistent with an *in situ* mechanism for core formation, HIV-1 particles with functional MA–CA cleavage but defective CA–SP1 cleavage exhibit a layer morphology^33^,^34^, indicating that the CA–SP1 layer is stable after cleavage of the MA–CA linkage. The proteolytic processing of the MA–CA cleavage site is faster than the processing of the CA–SP1 cleavage site^35^. As a result, one would assume that *in vivo* most of the CA layer will be released from the MA prior to CA–SP1 cleavage. At this stage, the N termini of the CA fold into the mature position^31^, which is thought to induce the formation of the mature CA lattice^31^. The morphology of the remaining CA–SP1 layer is consistent with the structure of the mature CA lattice^34^, which also supports an *in situ* change in the lattice organization of CA from the immature to the mature structure. Given that the CA–SP1 layer and the mature CA lattice, in the form of the core, are highly stable, any cleavage of SP1 is unlikely to disrupt the maturing CA layer; this then indicates that it is highly unlikely that most of the CA would dissociate from the lattice prior to core formation.

Our tomographic visualization of mature cores reveals significant defects in the hexagonal lattice structure of the mature CA (Supplementary Fig. 3), consistent with previous studies by Yu *et al*.^19^ Along with the canonical CA hexamers and pentamers expected in mature cores^14^,^36^, and directly resolved in our tomographic analyses (Fig. 3f, Supplementary Movie 2), we present clear evidence for a number of packing defects, including CA with heptameric geometry and numerous instances of cracks and gaps in the lattice structure. Non-diffusional phase transitions result in the formation of highly strained materials with significant defects as a result of the geometrical inconsistency between the precursor and product lattices^37^; such a transition can explain the origin of the extensive defects found in the cores. These stresses are also likely the driving force behind the curling of the CA sheet during core formation, observed both after complete release from the membrane^19^ and in the partially rolled core intermediate we report here (Fig. 6a,b,e,f and Supplementary Fig. 2).

By applying sub-tomogram averaging to our MA lattice images, we derived a map with sufficient resolution to observe the thin web-like structure of MA molecules that surround large hexagonal voids. The lattice parameters of the MA lattice, observed *in vivo* in this study, and of the MA lattices formed on an artificial membrane with lipid composition designed to mimic the cellular membrane^9^ are similar. However, our map showed significantly larger hexagonal voids than the *in vitro* lattice, suggesting different MA subunit organizations between these two lattices (Fig. 4). We suggest that in our structure, the MA trimers^22^,^23^ form hexamers by interacting through their tips; four residues known to be involved in Env incorporation^38^,^39^,^40^ are found at the tips of the trimers, and thus would be found in the proposed interface region. These results support the recently published model for Env incorporation by non-specific interactions with MA^11^. However, this type of array also minimizes the available area for interaction between the trimers; it is likely that these interactions stabilize the lattice poorly, producing an extremely fragile structure. This may explain why in most cases the MA lattice was found only as a patchwork of loosely connected small tiles (Fig. 3a), and was not visible on highly curved regions of the membrane of the multi-core containing structures.

Finally, release of the CA lattice from the membrane also likely releases constraints that promote membrane curvature. In regular virions, this has little effect; however, in the multi-core structures, which have a significantly lower surface to volume ratio, the natural curvature of the membrane is lower. Since the pool of available CA exceeds the amount necessary to form a mature core^41^, it is likely that any remaining cleaved CA dissociates into the interior of the virion either as free CA units or as small sheets, consistent with previous observations^19^,^41^. While there are still many quantitative aspects of the process of maturation that remain to be explained, our study allows us to integrate a number of previous observations into a single model for virion assembly and maturation. This new model, of a non-diffusional phase transition from immature Gag to mature CA lattice, represents a significant shift in our understanding of how HIV-1 virions assemble and mature during the infection process, and may elucidate novel therapeutic targets for preventing virion maturation and subsequent infectivity.

## Methods

### Viruses

HIV-1 BaL was produced from chronically infected SupT1-CCR5 CL30 cell line (AIDS and Cancer Virus Programme, Biological Products Core, CLN #204). The non-infectious strain HIV-1 NL4-3 Env^neg^, which contains a frameshift mutation and premature stop codon within the Env gene^42^, was produced from a subclone of the BC7.6.1A10 cell line, a CD4-negative derivative of SupT1, following transduction of BC7.6.1A10 cells with VSV-G-pseudotyped HIV-1 NL4-3 Env^neg^ and subsequent limiting dilution cell cloning and selection for a single-cell clone (CL. 60) producing high levels of virus (Del Prete *et al*., manuscript in preparation). Virions were purified from cell supernatants by sucrose density gradient centrifugation^43^.

Purified HIV-1 BaL virions were inactivated by a 30 min incubation at 4 °C in PBS containing 2% paraformaldehyde, which has been shown to leave core structure unaltered^44^. NL4-3 Env^neg^ samples were not inactivated. Virion pellets were stored −80 °C prior to sample preparation for cryo-electron microscopy.

### Grid preparation for cryo-electron microscopy

HIV-1 BaL or NL4-3 Env^neg^ virions were thawed on ice and resuspended with ~20 μl TNE buffer (10 mM Tris pH 7.4, 150 mM NaCl and, 1 mM EDTA), and 8 μl suspension of protein-A conjugated 10 nm gold particles (Utrecht University, Netherlands). Each specimen was prepared by transferring a 2.5 μl droplet of virion suspension onto a plasma-cleaned 200 mesh Quantifoil R2/2 grid (Quantifoil, Jena, Germany). Cryo-TEM grids were prepared by plunge freezing using a Vitrobot Mark IV (FEI). Grids were blotted for 6 s at room temperature and ~99% humidity, followed by plunge freezing into liquid ethane cooled by liquid nitrogen and transferred for storage into liquid nitrogen.

### Cryo-electron tomography

Cryo-electron tomography was performed as described^45^ using a Polara Tecnai G2 Transmission electron microscope (FEI Company, Hillsboro, OR, USA) equipped with a 4 × 4 K CCD camera at the end of an energy filter (Gatan Inc., Pleasanton). Tilt series were recorded with either 1° or 2° increments between ±60° with a total dose of 150–180 e^−^/Å^2^. Tilt series were imaged with a 200 kV beam at a −3 μm defocus and × 34 K magnification, with a pixel size of 3.9 Å at the sample plane. The pixel size at the reported imaging conditions was measured using catalase crystal and cross grating calibration grids as standards (EMS, Hatfield, PA, USA).

### Tomographic reconstruction and image processing

Fiducial-based alignment of the tilt series was performed by automatic tracking of the gold nanoparticles embedded in vitreous ice^46^. For visual inspection and manual segmentation, the pixels of the tilt were binned by 4, reconstructed into tomograms using Weighted Back Projection^47^, then low pass filtered to 0.45 Nyquist frequency. Regions-of-interest in the tomograms that were used for sub-tomogram averaging or for power spectrum calculation were reconstructed from the unbinned tilt series using 150 iterations of the simultaneous iterative reconstruction technique^48^. A total of 184 HIV-1 BaL and 79 Env^neg^ membrane-enclosed multi-core containing structures were analyzed, and 450 HIV-1 BaL and 165 Env^neg^ regular virions were analyzed through tomographic analysis.

Power spectra of regions-of-interest (areas showing the same lattice over several tomographic slices) were obtained by calculating the absolute value of the Fast Fourier Transform for each slice and averaging the resulting patterns. The missing wedge adds a non-homogenous smooth background to the power spectrum pattern with preferred stronger background in the direction parallel to the tilt axis. This background was estimated from slices of the tomogram with an empty field of view consistent with the vitreous ice above and below the region-of-interest. The resulting background was subtracted from the average power spectrum pattern of the region-of-interest. The spectra in Fig. 3b,c were derived from the regions designated by yellow rectangles in Figs 3a and 4b. The lattice spacing obtained from the power spectra of the MA lattice in Figs 3a,d and 4b were the same.

### Sub-tomogram averaging

Tomogram volumes were boxed using the grid-threading Monte Carlo searching method^49^. Using the local maximum clustering method that we have described in detail before^50^, major clusters were identified. The cluster with the greatest C6 symmetry was chosen and used as the template for further alignment of subvolumes. This sequence of alignment and clustering steps was repeated until no further changes in alignment were observed. The final major cluster is the averaged volume that we reported here.

### Quantitative structural analysis

The surface areas of the membranes and the number of cores in each object were calculated starting with the tomograms. The surface areas were calculated by treating these objects as ellipsoids with one semi-principal axis along on the *z*-axis system of the tomogram. The two other semi-principal axes were measured on the slice with the widest cross-section. Some multi-core structures had the appearance of large tubes with spherical ends. In these cases, the length and width of the tubes was measured and the area was calculated accordingly. The number of cores and areas were calculated for 42 multi-core structures and 67 virions from the HIV-1 BaL tomograms, and for 40 multi-core structures and 52 virions from the Env^neg^ tomograms. To visualize the results of these measurements, scatter plots of the surface area, the number of cores and the surface area per core were obtained (Supplementary Fig. 1).

### TEM and FIB-SEM of cell culture samples

In preparation for 2D TEM and FIB-SEM, HIV-infected CD4^+^ T cells were fixed, pelleted and resin-embedded^51^. Briefly, the cell pellets were chemically fixed with aldehydes and osmium tetroxide, stained with uranyl acetate, dehydrated through graded ethyl alcohol and embedded in epoxy resin. Hundred-nanometer thick slices of the resin-embedded cell pellet were evaluated by TEM imaging to obtain projection images using a Tecnai 12 microscope (FEI Company). FIB-SEM imaging was performed using a Zeiss NVision 40 microscope, with the SEM operated at 1.8 keV landing energy, and backscattered electrons were recorded at an energy-selective back-scattered electron detector with a grid voltage of 1,000 V. A patterned Pt–C double protective layer was deposited on the region-of-interest to protect the area from FIB damage and to accurately advance the FIB during milling^51^. The user interface employed was ATLAS 3D from Carl Zeiss; the imaging parameters used were 1 μs dwell time, line average 4, pixel sampling set at 5 nm, and *z* slices were set at 10 nm. The stack of images generated during an image acquisition run were first binned in *x* and *y* to yield isotropic voxels of 10 nm. They were then inverted, tilted and trimmed as required using the IMOD package^47^, and aligned by cross-correlation^52^. The 3D FIB-SEM data set was manually segmented with Slicer 3D (ref. 53); www.slicer.org) to highlight the cores within the region-of-interest.

## Additional information

**How to cite this article:** Frank, G. A. *et al*. Maturation of the HIV-1 core by a non-diffusional phase transition. *Nat. Commun.* 6:5854 doi: 10.1038/ncomms6854 (2015).

## Supplementary Material

Supplementary Figures Supplementary Figures 1-3

Supplementary Movie 1 The movie demonstrates the formation of a core by detachment and rolling of a CA sheet directly from the immature Gag assembly.

Supplementary Movie 2 The movie runs through the slices of the tomogram, showing the overall structures of the core, and the CA arrangement as revealed in some of the raw slices and by the automatic segmentation. Several hexamers, a pentamer, and a heptamer are clearly visible.
