# Supplementary material for: Maturation of the HIV-1 core by a non-diffusional phase transition
Source: Nat Commun. 2015 Jan 8;6:5854. doi: 10.1038/ncomms6854 (PMC4288007; doi:10.1038/ncomms6854)
Supplement: Supplementary Figures — 1-3 [file ncomms6854-s1.pdf]

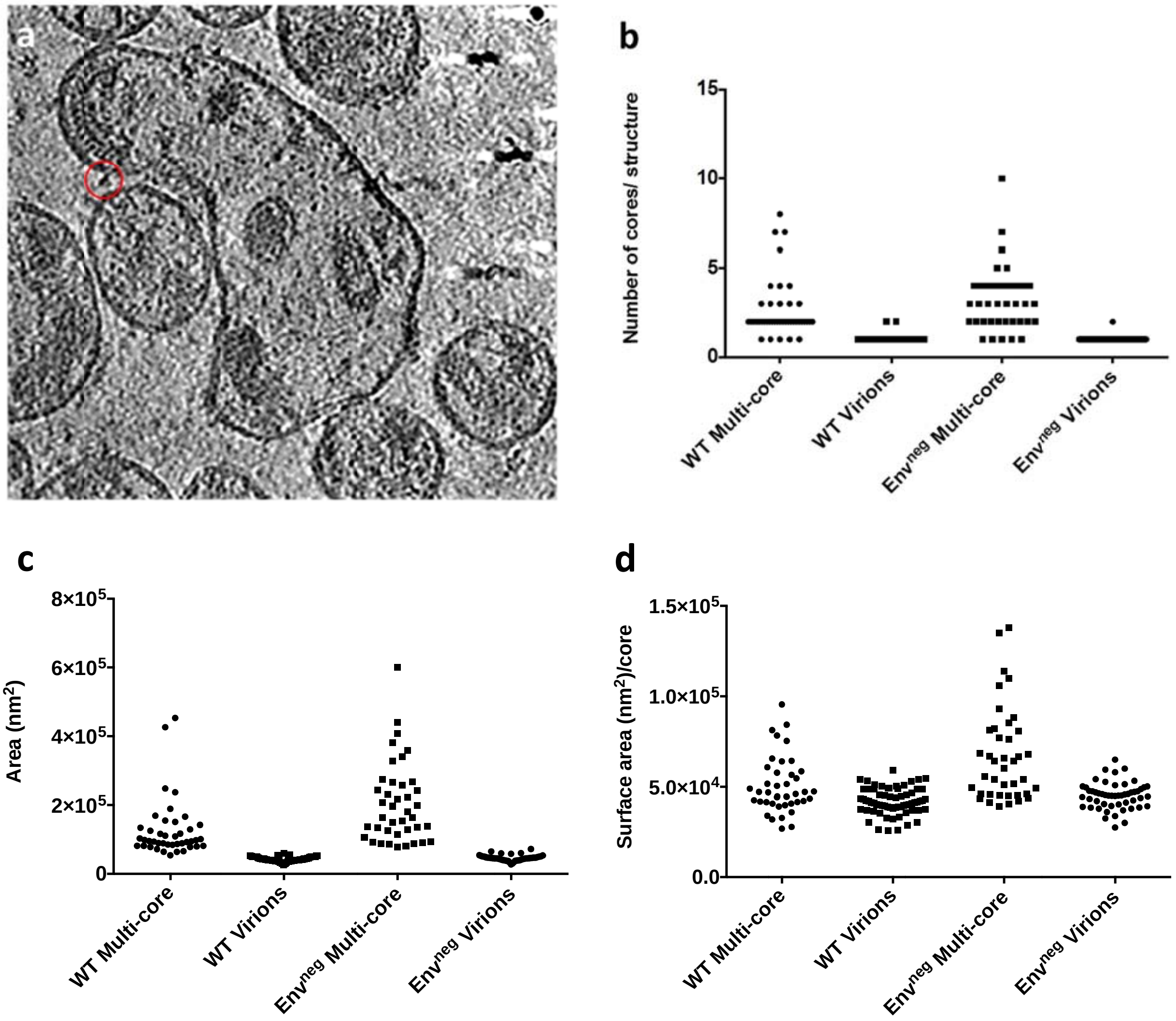

Supplementary Figure 1: (a) Gag in a HIV-1 BaL multi-core membrane-enclosed structure. A slice through a tomogram of a HIV-1 BaL multi-core membrane-enclosed structure with three cores and a Gag assembly (top left). A protein consistent with Env is designated by a yellow circle. Scale bar is 50 nm. (b-e) Scatter plots of various quantitative features of virions and multi-core structures from HIV-1 BaL and Env^{neg} cultures. (b) Number of cores present in virions and multi-core containing structures. (c) The surface area of multi-core structures and virions. (d) The surface area of the membrane for multi-core structures and virions, divided by the number of cores present inside the object.

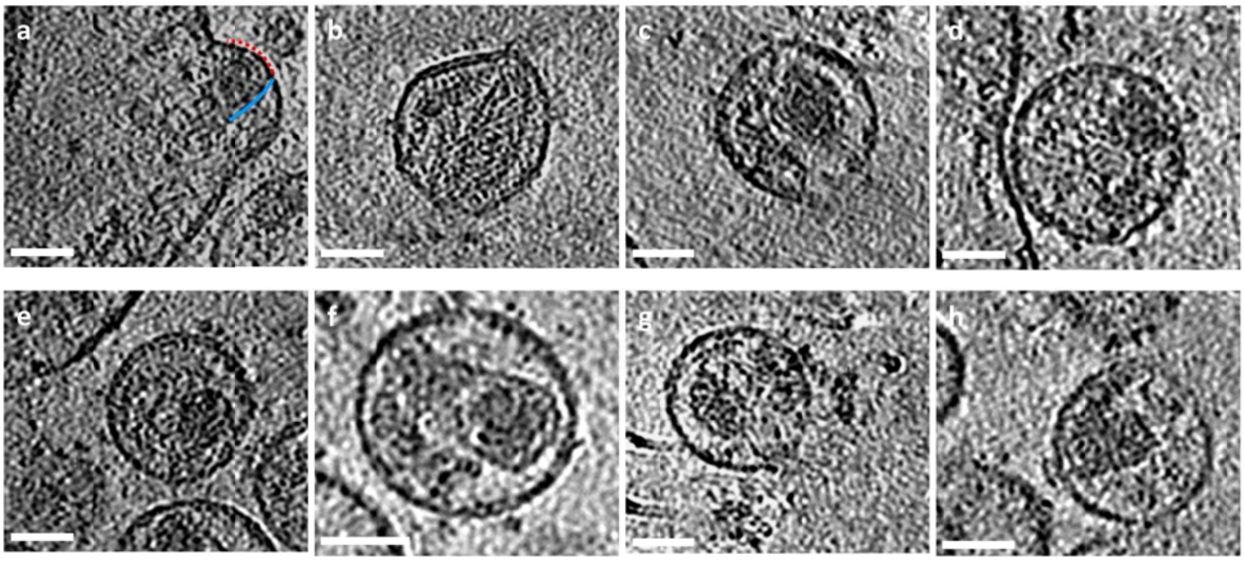

Supplementary Figure 2: Stages of core formation on the membranes of multi-core membrane-enclosed structures and regular virions. (a) A slice through a tomogram of a forming core on the membrane of a multi-core membrane-enclosed structure. A small part of the Gag is still attached to the membrane (red dashed curve), and an array of high density points consistent with CA is found in direct continuation of the immature CA layer (light blue curve). (b-h) Examples of cores in intermediate stages of formation within normal-sized virions. The tomographic slices presented here were selected to show a cross section through the core perpendicular to the long axis. These sections have the membrane bound region and the core in the same plane. Scale bars are 50 nm.

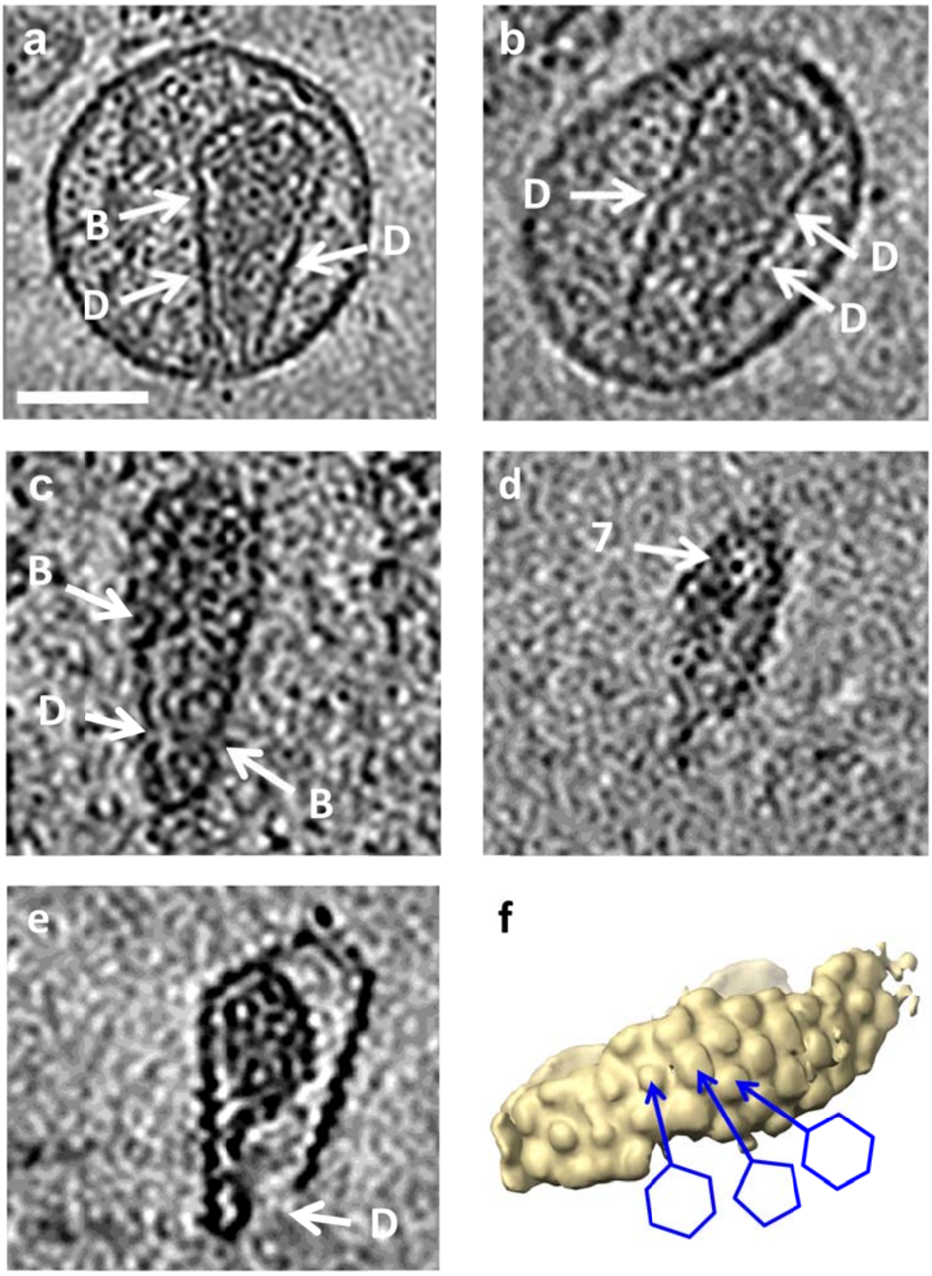

Supplementary Figure 3: Gallery of defects often found in viral cores. (a-d) Examples of defective cores show bumps (B), discontinuities (D) and 7-fold geometry (7), as well as hexameric and pentameric arrangement of CA in cores. (e) A slice through a tomogram of a fully formed core with a distinct gap at the end (arrow). (f) Segmentation and rendering of part of the core in (e) reveals the arrangement of CA oligomers. Some oligomers have hexameric geometry and one has a pentameric geometry. Scale bars are 50 nm.
